# Blood-Based Analysis of Circulating Cell-Free DNA and Tumor Cells for Early Cancer Detection

**DOI:** 10.1371/journal.pmed.1002205

**Published:** 2016-12-27

**Authors:** Klaus Pantel

**Affiliations:** Institute of Tumor Biology, University Medical Center Hamburg-Eppendorf, Hamburg, Germany

## Abstract

In a Perspective, Klaus Pantel discusses prospects for use of cell-free DNA and circulating tumor cells in cancer detection.

## Introduction

Analysis of circulating cell-free DNA (cfDNA) or circulating tumor cells (CTCs), also known as liquid biopsy, has received substantial attention in recent years owing to the potential benefits for detecting tumors and informing treatment [1]. cfDNA is released into the blood by apoptotic and necrotic cells, and cancer patients have increased concentrations of cfDNA [2]. CTCs are released by primary tumor lesions into the blood and eventually home to distant organs such as liver, bone, lungs, or brain. Building on groundbreaking work by Kinzler, Vogelstein, and colleages [3], novel technologies now enable detection of tumor-associated mutations in cfDNA. The choice of cfDNA sequencing technology depends on the fraction of circulating tumor-derived DNA (ctDNA) within the pool of cfDNA, which is usually much higher (>5%–10%) in patients with advanced disease compared to patients at earlier disease stages (<1%) [4,5]. Technologies that can detect minute amounts of ctDNA have been developed, but these require proof-reading measures to avoid artefacts [6]. Genomic analyses of single CTCs require whole genome amplification to obtain sufficient amounts of DNA, which needs to be controlled well to minimize bias [7]. Consequently, the detection of very low amounts of ctDNA and CTCs in blood samples from early-stage cancer patients, and assessment of the possible clinical significance of the resulting findings, is still a challenge [4,5].

Besides CTCs and ctDNA, liquid biopsy analyses can also focus on microvesicles (particularly exosomes), cell-free microRNAs, and blood platelets, which also provide interesting information on molecular features of a tumor lesion and might, therefore, have the potential to contribute to early detection of cancer [8–10]. Moreover, like CTCs, exosomes are biomarkers with a biological function in metastatic development [11]. However, the present discussion focuses on CTCs and ctDNA, which are currently the most prominent targets of liquid biopsy analyses in cancer patients.

## cfDNA in Patients with Early-Stage Cancer

As an example, Fernandez-Cuesta and colleagues [12] recently used a pipeline specifically designed to accurately detect sequence variants present at very low fractions in cfDNA from patients with small-cell lung cancer (SCLC), addressing TP53 mutations, which are known to occur in the majority of cases of SCLC [13]. TP53 mutations were detected in only 35.7% of early-stage cases, indicating limited sensitivity as marker for detection of early SCLC. More sensitive technologies or the addition of other cancer-specific mutations, or combinations thereof, might in the future be able to increase the sensitivity of liquid biopsy in this type of setting. Moreover, quite large plasma volumes could be required for early cancer detection, because the ctDNA concentration in early-stage lung cancer patients can be as low as one genome equivalent in 5 ml blood [6]. Bettegowda and coworkers used digital PCR-based technologies to evaluate the usefulness of ctDNA for detection of tumors in 640 patients with various cancer types [4]—ctDNA was detected in only 48%–73% of patients with localized colorectal cancer, gastroesophageal cancer, pancreatic cancer, and breast adenocarcinoma. Again, these detection rates are not satisfactory for early cancer detection because false negative determinations are likely to be common.

## Cancer-Associated Mutations in Cancer-Free Individuals

In addition to the need for higher sensitivity, the specificity of cfDNA measurements also faces serious challenges. Recent reports have shown that cancer-associated mutations are not restricted to cancer patients. For example, TP53-mutated cfDNA fragments were observed in 11.4% of 123 matched noncancer controls [12], and Krimmel and colleagues demonstrated very low levels of TP53 mutations in the peritoneal fluid and peripheral blood of women with benign ovarian lesions [14]. Likewise, leukemia-associated mutations have been shown to occur with increasing age and, although such mutations pose a statistically significant risk for a person to develop leukemia, most individuals (>90%) with these mutations never developed leukemia during their lifetime [15]. Because the presence of cancer-associated mutations in cfDNA might not necessarily indicate that the individual tested has cancer or will develop cancer, the risk of substantial anxiety for patients and unnecessary medical interventions is substantial.

The findings of such studies appear to reflect general principles that challenge the promise of ctDNA analysis for early cancer detection. Genomic alterations, such as those in the *BRAF*, *RAS*, *EGFR*, *HER2*, *FGFR3*, *PIK3CA*, *TP53*, *CDKN2A*, and *NF1/2* genes, all of which are considered hallmark drivers of specific cancers, can also be identified in benign and premalignant conditions, occasionally at frequencies higher than in their malignant counterparts [16]. For example, *BRAF* V600E mutations are paradoxically more frequent in benign nevi (∼80%) than in dysplastic nevi (∼60%) or melanomas (∼40%–45%) [17]. Driver mutations also occur in nonmalignant conditions, such as *FGFR3* mutations in seborrheic keratosis [18].

## CTCs for Early Detection of Cancer

Cancer patients harbor CTCs in their bloodstream that can be detected at very low concentrations using advanced technologies [19]. After an enrichment step that increases the concentration of CTCs by several log units, CTCs can be positively or negatively enriched on the basis of biological properties (i.e., expression of protein markers) or on the basis of physical properties (i.e., size, density, deformability, or electric charge) [19]. Positive or negative CTC enrichment can also be combined in the same device. CTCs are released early during tumor development and have been found in patients with small primary tumors (e.g., breast cancer) [20]. Thus, detection of CTCs rather than cfDNA might be an alternative approach to early cancer identification. In patients with chronic obstructive pulmonary disease (COPD) who have an elevated risk of developing lung cancer, Ilie and colleagues reported that CTCs could be detected in 3% of COPD patients [21]. Annual surveillance of CTC-positive COPD patients by CT screening detected lung nodules one to four years after CTC detection, leading to prompt surgical resection and histopathological diagnosis of early-stage lung cancer, while no CTCs were detected in control smoking and nonsmoking individuals with normal lung function. These encouraging preliminary findings need to be validated in larger cohorts and other tumor types with similar conditions conferring an increased risk of cancer.

## Current Challenges of cfDNA Analyses

Achieving acceptable levels of sensitivity and specificity for early cancer detection will require further technical advances. Extensive research is required to identify possible combinations of cancer-specific mutations and define potential quantitative thresholds to avoid overdiagnosis. Based on the findings to date, it can already be envisaged that DNA sequencing needs to be broad, so as to encompass considerable tumor heterogeneity, and deep, to detect minute amounts of ctDNA fragments in the milieu of extensive genetically normal cfDNA. Nonmalignant conditions that can lead to the death of normal cells may also lead to a further dilution of ctDNA molecules and hamper quantitative evaluations. Very sensitive technologies that allow the detection of less than 0.1% of ctDNA in blood plasma (e.g., digital droplet PCR or cancer personalized profiling [CAPP] by deep sequencing methods) have been developed and applied to cfDNA analyses in patients with various forms of cancer [4,6,22], but the key biological limitation might be the number of genome equivalents present in blood samples from early-stage cancer patients [5].

## Current Challenges of CTC Analyses

The potential use of CTCs for early cancer detection faces similar challenges of sensitivity and specificity. DNA sequencing of single CTCs has been successfully performed by several groups, but the whole genome step required to obtain sufficient amounts of DNA requires standardization [7]. RNA sequencing of CTCs has also become possible [23], but this approach requires rapid CTC capture technologies to avoid RNA degradation. Moreover, the analysis of larger blood volumes might be required to obtain sufficient amounts of CTCs, which is a limitation for a cancer screening method. Conditions that may lead to nonspecific findings in noncancer patients, such as the release of epithelial cells into the bloodstream of patients with inflammatory bowel diseases [24], need to be explored to avoid false positives. Nevertheless, CTC capture should also permit functional studies in xenograft models [25,26] and the development of cell lines as models for in-depth investigation of CTC biology and drug testing [27].

## Conclusion and Perspectives

Studies evaluating new approaches to early cancer detection usually begin with comparisons of small cohorts of cancer patients with controls (healthy individuals or patients with benign diseases). Subsequent investigations will typically involve larger study populations and extended follow-up times and require substantial financial resources. Focusing on population groups at an elevated risk of developing cancer (e.g., COPD patients or patients with a well-known genetic predisposition to breast or colon cancer) is a good strategy to speed up the process of testing and validation of new approaches and technologies. In contrast, large population-based screening efforts may reveal tumor lesions that cannot generally be cured by current therapies (e.g., pancreatic cancer) or that have an indolent disease course in many patients (e.g., prostate or thyroid cancer) and where the need for therapy is not clear.

Another important clinical application is early detection of minimal residual disease (MRD) in patients with resected primary tumors. Postsurgical surveillance of MRD by cfDNA analysis has enabled investigation of clonal evolution in colorectal cancer patients [22,28]. Current clinical trials are focusing on stratification and monitoring of therapies in cancer patients using CTC or cfDNA analyses. For example, in breast cancer, the METABREAST trial is assessing whether CTC counts are helpful in the decision about whether chemotherapy or less aggressive endocrine therapy is needed, while the DETECT-III trial is evaluating whether patients with HER2-negative primary tumors but HER2-positive CTCs will profit from anti-HER2 therapy with lapatinib [29]. Regarding the use of cfDNA for early cancer detection, enormous resources are required to identify genomic aberrations specific and sensitive enough to detect early malignant lesions in large cohort studies.

Finally, verification of the findings of approaches involving liquid biopsy will call for acquisition of information on the putative location of the occult lesion to select the appropriate imaging modalities or other diagnostic means prior to planning appropriate therapies. In this context, the detection of tissue-specific gene expression in CTCs [30] might be a promising approach.

## Abbreviations

CAPP: cancer personalized profiling
cfDNA: circulating cell-free DNA
COPD: chronic obstructive pulmonary disease
CTC: circulating tumor cell
ctDNA: circulating tumor-derived DNA
MRD: minimal residual disease
SCLC: small-cell lung cancer
